# P54. Possible role of MDSC for the induction of tumour-specific T cells following LRAST

**DOI:** 10.1186/2051-1426-2-S2-P28

**Published:** 2014-03-12

**Authors:** P Rose, JR Kovács, RA Hatz, H Winter, NK van den Engel

**Affiliations:** 1 Klinikum der Universität München-Großhadern, Klinik für Allgemeine Viszeral- Transplantations- Gefäß- und Thoraxchirurgie, Munich, Germany

## Background

*L*ymphodepletion with subsequent *r*econstitution and *a*ctive-*s*pecific *t*umour cell vaccination (LRAST) during the homeostatic proliferation is a promising approach in cancer immunotherapy. While tumour specific response is evident under LRAST regime, simultaneous induction of potentially immunosuppressive immature myeloid cells (MDSC) may limit the therapeutic efficacy. Following LRAST MDSC can potentially be derived from the host or the donor cell pool. Aim of the study was to analyse the role of granulocytic (G-MDSC: CD11b^+^ Ly6G^+^ Ly6C^+^ ) and monocytic (Mo-MDSC: CD11b^+^ Ly6G^-^ Ly6C^+^) MDSC and to track their fate in the recipient mouse following LRAST.

## Material and methods

Female C57BL/6 (Ly5.2) mice were challenged with tumour by subcutaneous injection of 5×10^4^ vital D5 melanoma cells and lymphodepleted 3 days later by i.p. application of 200mg/kg cyclophosphamide. After 24 h mice were reconstituted with i.v. injection of 2×10^7^ splenocytes from naïve B6.SJL-PtprcaPepcb/BoyCrl (Ly5.1) mice followed by vaccination with 1×10^7^ irradiated mGM-CSF-producing D5G6 cells. Mice lacking tumour challenge, lymphodepletion, or tumour vaccination were included as controls. Splenocytes, peripheral blood cells and lymph node cells were analyzed by 8 color flow cytometry at days 3, 10 and 23. Induction of tumour-specific T cells in tumour vaccine draining lymph nodes was evaluated in vitro by tumour specific IFN-γ secretion assays.

## Results

A pronounced increase in host G- and Mo-MDSC was observed in spleens of both lymphodepleted and vaccinated groups between day 3 and 10. A decrease of T-cell subsets, including memory T-cells, and natural killer (NK) cells was detected at day 10. Consecutively to the rapid decline of MDSC after day 10 a numeric gain in the T-cell fraction was observed. In contrast NK cells remained at a low level until day 23. Donor G- and Mo-MDSC were detected at low frequencies at all time points. Donor MDSC as identified by Ly5.1 slightly increased between day 3 and 10 followed by a decline after day 10. The reconstituted cell fraction predominantly consisted of T-cells (up to 29.7% of leukocytes). Helper and effector T-cells were continuously increasing during the observed period of time while memory type T-cells (CD8^+^ Gr1^+^) were being suppressed.

## Conclusions

The elevated numbers of MDSC at day 10 after treatment may inhibit the induction of tumor specific T cells. Therefore depleting MDSC in the recipient mouse with anti-Gr1 antibody in the early phase of the LRAST treatment could be a promising approach to improve anti-tumour efficacy.

